# Optimization of inter-row spacing as influenced by genotype by environment interaction (G×E): A dataset on frontiers of malt barley *(Hordeum Distichum L.)* productivity

**DOI:** 10.1016/j.dib.2021.107542

**Published:** 2021-11-05

**Authors:** Amare Assefa Bogale, Anteneh Agezew Melash, Awoke Wasae Argaw

**Affiliations:** a College of Agriculture and Environmental Science, Department of Horticulture, Debark University, P.O.Box, 90, North Gondar, Debark, Ethiopia; b College of Agriculture and Environmental Science, Department of Plant Science, Debark University, P.O.Box, 90, North Gondar, Debark, Ethiopia

**Keywords:** Dataset, Growing environment, Phenological traits, Malt barley varieties, Yield components, Yield

## Abstract

Ethiopia is one of the major producers of barley in Sub-Saharan Africa and has a growing malt beverage sector. Yet, despite a favourable bio-physical environment, malt barley productivity is much lower than the potential yield due to traditional land management systems, poor agro-technical crop management practices, and lack of high-quality seeds. Field experiments were therefore conducted to evaluate the improved malt barley varieties under a range of inter-row spacing in two different growing locations, for their yield and other associated traits. The treatments were arranged in randomized complete block design consisting of six malt barley varieties (i.e. Holker, HB1963, Sabine, Ibone174/03, EH1847, and Freygebse), and three levels of inter-row spacing (i.e. 20, 25, and 30 cm) in two divergence growing locations (i.e. Miligebsa and Kino). Agronomic practices such as weeding, fertilizer application, harvesting, and threshing were uniformly applied for all experimental units. The data presented under this dataset article includes phenological traits (i.e. days to 50% heading, and days to 90% physiological maturity), seed yield and yield components (i.e. seeds spike^−1^, spike length, number of effective tillers, 1000-seed weight, total aboveground biomass, and straw yield). All the collected data were subjected to a statistical analysis software package using the general linear model (GLM) procedure of the SAS 9.2 version. This dataset article therefore provides information about how optimization of inter-row spacing varied variety to variety. Additionally, it provides how the environment diverges the efficiency of inter-row spacing for maximum potential yield. Hence, this information can allow other researchers to review the supplement data, methods, and make detailed analysis, which possibly giving rise to new lines of inquiry. This can also give rise to new collaborations and boost the reputation of the present research results within the scientific community. This dataset article is aimed to provide a dataset collected from an intensive malt barley field experiment for public use and to make it available to everyone around the subject matter to use as they wish.

## Specifications Table

**Table:** 

Subject area	Agriculture
Specific subject area	Agronomy
Type of data	Table and Graphs
How data was Acquired	Various data types, such as agronomic and phenological traits were collected by using measurement under field conditions.
Data format	Analyzed mean data and clean raw data
Parameters for data collection	Six improved malt barley varieties, and three levels of inter row spacing (i.e. 20, 25, and 30 cm), were considered and tested under two mega environments. The tested experimental sites were fertilized with Nitrogen and phosphorus as Di-ammonium phosphate (DAP), and Urea (46-0-0), was uniformly supplied into all experimental plots at a rate of 46 kg ha^−1^ of nitrogen (N) and 20 kg ha^−1^ of phosphorus (P), under favorable weather to minimize nitrogen loss. The mean annual rainfall and temperature during the cropping season in the study district was 974 mm, 12.4 °C, respectively.
Description of data collection	Phenological and agronomic based data such as days to 50% heading, and days to 90% physiological maturity, plant height, seeds spike^−1^, spike length, effective tiller numbers, 1000-seed weight, aboveground biomass yield, straw yield and grain yield were collected by measuring traits at the specified times in the experimental sites. The agronomic traits were recorded from 10 randomly selected plants while grain, straw and aboveground biomass yields were collected on a plot basis. Instruments such as portable hanging weighing scale (Model: 23510S (SHFSB-0403)) was used to measure the aboveground biomass yield (t ha^−1^), a digital electronic sensitive balance was used to measure final harvestable yield and 1000 seed weight, and measuring tape was used to measure the spike length and plant stature.
Data source location	Debark University (DKU), northern Gondar, Ethiopia, is the owner of the data. DKU is located in Amhara regional state, at 13°30′N and 39°29′E with an elevation of 2210 meters above sea level. The experimental sites are located at 13°4′51″–13°4′52″ N and 37° 53′17″–37° 53′19″E with an elevation of 2717 m.a.s.l and 13°1′4″–13°11′42″N and 37° 59′2″–37° 59′3″E with an elevation of 3153 m.a.s.l, ***Kino***, and ***Milligebsa*** respectively.
Data accessibility	With article and the raw data is deposited in the Mendeley dataset repository available at: https://data.mendeley.com/datasets/2sp38s8y3b/1
Related research article	**Amare A., Ketema N., Awoke W., and Shegaw H., (2021)** Response of Malt Barley *(Hordeum Distichum L.*) Varieties to Different Row Spacing under Contrasted Environments of North Gondar, Ethiopia. International Journal of Agronomy, vol. 2021, Article ID 6696470, 12 pages. https://doi.org/10.1155/2021/6696470

## Value of the Data

• This dataset provides valuable information on optimization of inter-row spacing, and its importance, as each crop needs a certain amount of room for its roots and leaves to maximize its productivity. Hence, maintaining proper row spacing can help in improving the air circulation around and in between the plants, which helps to prevent diseases from spreading, especially in a wet and humid environment. This information, therefore, can help malt barley producing farmers to minimize the cost of chemical spray to protect pests and diseases cause by narrow spacing, and for agronomists, and agricultural development agents to consider varietal differences in spacing, disease pressure, and weed control options when sowing malt barley varieties in narrow row spacing.
• This dataset article can be further used for plant breeders to elucidate the association between malt barley seed yield and phenotypic traits under narrow and wider row spacing cropping systems for the development of predictive tools useful in prescriptive breeding efforts tailored to production systems. This could enable an increase in the rate of genetic gain and malt barley producing farmers’ profitability, and will allow to improve research operational efficiency among scholars.
• The dataset in this article provides information about defining and optimization of row spacing under wet and marginal environments. The dataset further illustrates the factors that affect the determination of the optimum raw spacing for a specific location.

## 1 Data Description

**Fig. 1:**
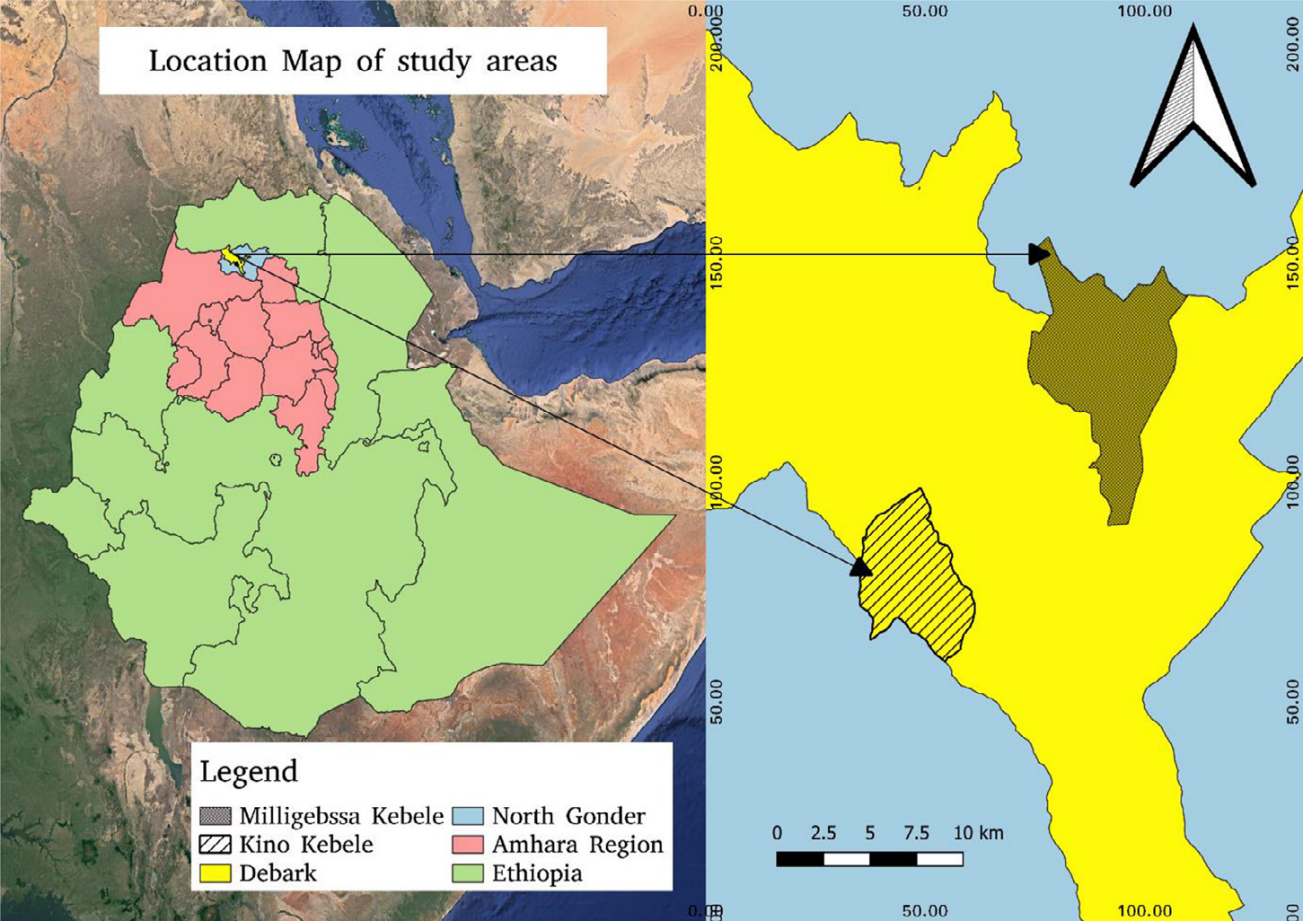
Map of the study area: Amhara National Regional State in Ethiopia, North Gondar in Amhara region, Debark district in North Gondar, *Kino* and *Miligebsa Keble* in Debark district.

**Fig. 2:**
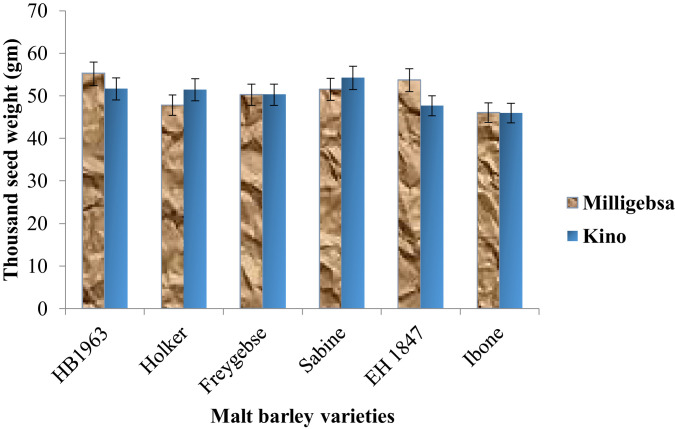
The response of thousand seed weight to the interaction effect of malt barley varieties and growing locations.

**Fig. 3:**
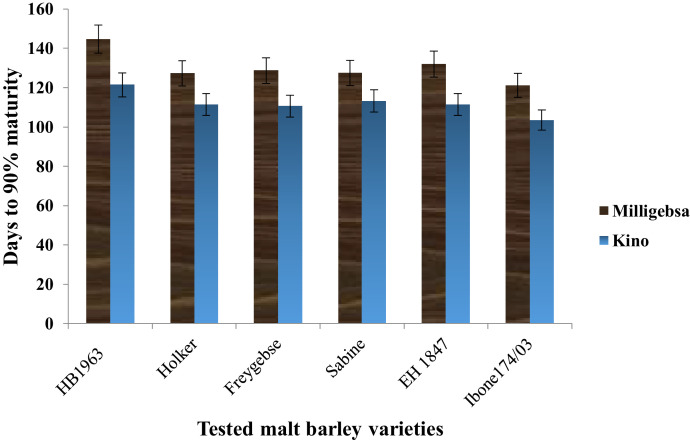
Illustrated that the length of maturity period influenced by growing location and divergence in adaptation level of malt barley varieties.

**Fig. 4:**
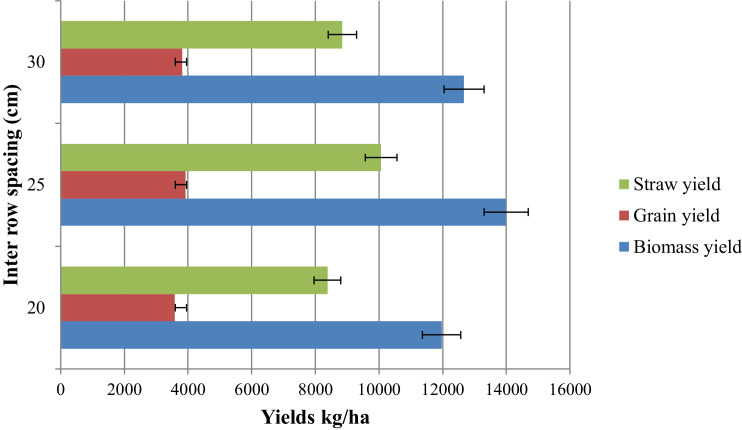
This figure illustrated that, the positive benefit of increased yields with increasing the inter-row spacing up to around 25 cm after which there is little apparent reduction in grain, biomass and straw yields.

**Fig. 5:**
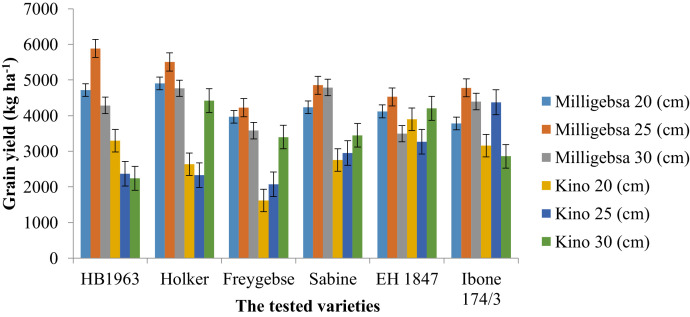
A grain yield of malt barley varieties as a function of divergence in growing location under a range of inter-row spacing. Further, this figure illustrated that optimization of inter-row spacing is significantly dictated by variation in growing location and crop varieties.

The principal farm activities such as varietal selection, change in row spacing, and selection of suitable growing environments are key elements in the production of quality malt barley grains. This dataset article is, therefore describes the factors that affect agronomic traits, yields, and phenological traits of malt barley varieties (Fig. 2, Fig. 3, Fig. 4, Fig. 5). Fig. 1 illustrates a map of the study areas. The data presented in Fig. 2 showed the interaction effect between malt barley varieties and inter-row spacing on thousands of seed weights. Fig. 3 provides an important interaction effect between inter-row spacing and varietal difference to influence the length of phenological periods such as the days to physiological maturity. The data presented in this article is fairly consistent for both growing locations and shows that narrower row spacing can improve grain yield, total aboveground biomass, and straw yields (Fig. 4). Variations in grain yield across tested varieties, growing location, and inter-row spacing gave interesting insights to consider the selection of suitable malt barley varieties and defining varietal-specific inter row spacing (as shown in Fig. 5). Table 1 contains the passport data of the tested malt barley varieties such as suitable agro-ecological zones, released year and realised centre. The soil physicochemical properties of both experimental sites (i.e. Kino and Milligebsa) are indicated in Table 2. Table 3 describes the analysis of variance (ANOVA) of the mean squares for the main and interaction effect of variety, inter-row spacing, and growing location on phenological traits, agronomic traits and yields including grain, total aboveground biomass and straw. This dataset article provides the raw data associated with each and individual repeat in both experimental conditions and thus, the raw data is deposited in the Mendeley dataset library (https://data.mendeley.com/datasets/2sp38s8y3b/1).

**Table 1:** Physicochemical properties of soil the experimental sites (Kino and Milligebsa).

No.	Physicochemical properties of soil	Kino	Milligebsa
1	Soil PH	5.50	5.32
2	Sand (%)	36	40
3	Silt (%)	35.28	37.28
4	Clay (%)	28.72	22.72
5	Texture class	Clay loam	loam
6	EC (ds/m)	0.12	0.21
7	Organic carbon (%)	2.84	6.51
8	Available P (PPM)	18.64	26.97
9	CEC (Cmol/kg)	35.7	32.44
10	Ex. K+(Cmol/kg)	0.51	0.15

*Source:* Gondar soil laboratory 2019.

**Table 2:** Passport data of malt barely variety used for the experiment (Ministry of Agriculture, 2016).

				Agro-ecological zone

Improved varieties	Released Year	Released at:	Grain yield (t/ha) during the release period	Altitude (masl)
Freygebse	2010	HARC	4.0	2300–3000
Holker	1979	HARC	2.4–3.1	2300–3000
Sabine	2011	KARC/HARC	2.5–3	2300–2800
EH1847	2011	HARC	3.5–4.0	2300–2800
Ibone 174/03	2012	HARC	3–5.7	2300–2800
HB1963	2016	HARC	3.5–6.0	Above 2300

**Key to abbreviations: HARC**; Holeta Agriculture Research Centre; **KARC**; Kulumsa Agriculture Research Centre.

**Table 3:** The analysis of variance (ANOVA) of the mean squares for the main and interaction effect of variety, inter row spacing and location on phenological, agronomic and yields.

		Phenological traits		Selected agronomic traits								
Source of variation	Df	DH	DM	PH (cm)	SPL(cm)	ETN	NKPS	TKW (g)	BY (kg/ha)	GY(kg/ha)	SY (kg/ha)	HI
Replication	2	2.9	10.1	55.5	0.2	359.7	128.744	33.4	3972286	23639.9	3437074	3.5
Location	1	11142.7^⁎⁎^	8983.6^⁎⁎^	2628.8^⁎⁎^	7.5*	46691.8^⁎⁎^	25.0^⁎⁎^	7.9^ns^	19783032*	54212960.5^⁎⁎^	1377536265^⁎⁎^	2423.1^⁎⁎^
Varieties	5	187.7^⁎⁎^	812.68^⁎⁎^	793.14^⁎⁎^	1.9^⁎⁎^	806.4^⁎⁎^	44.1713^⁎⁎^	128.3^⁎⁎^	247689^⁎⁎^	1944383^⁎⁎^	18270389^⁎⁎^	77.9^⁎⁎^
IRS	2	1.3^ns^	18.36^ns^	113.78^ns^	0.2 ^ns^	2491.7^⁎⁎^	3.6700 ^ns^	58.9^⁎⁎^	38081408^⁎⁎^	1079136^⁎⁎^	27291718^⁎⁎^	75.4^⁎⁎^
V × IRS	10	8.8^⁎⁎^	9.06^ns^	55.53^ns^	0.2^ns^	280.4*	3.0547 ^ns^	10.4^⁎⁎^	10534251^⁎⁎^	984825^⁎⁎^	6833453^⁎⁎^	31.7^⁎⁎^
L × V × IRS	10	6.8*	7.0^ns^	83.73^ns^	0.5^⁎⁎^	200.4^ns^	1.8304 ^ns^	1.608^ns^	10696897^⁎⁎^	1092314^⁎⁎^	5936810^⁎⁎^	12.8^⁎⁎^
Error	70	6.8	7.98	81.67	0.15	112.4	0.88	3.7	856034	37744	866859	3.4
R^2^		0.98	0.95	0.60	0.77	0.90	0.65	0.79	0.98	0.978	0.97	0.94

**Key to abbreviations:** IRS = Inter row spacing, DH = Days to heading, DM = Days to maturity, PH = Plant height, SPL = Spike length, ETN = Effective tiller, NKPS = Number of kernel per spike, TKW = Thousand kernel weight, BY = Biological yield, GY = Grain yield, SY = Straw yield, HI = Harvest index, DF = degrees of freedom; NS, * and ** = non-significant, significantly different at 5 % and 1%, respectively according to LSD test.

## 2 Experimental Design, Materials and Methods

The field experiments were conducted in two different growing locations, in North Gondar, Ethiopia (Fig. 1). At each experimental site, six malt barley varieties including ***Holker, HB1963, Sabine, Ibone174/03, EH1847, and Freygebse,*** were used as a test crop. These varieties are realised for their adaptability under a range of agro-ecological zones (Table 1). The six malt barley varieties were therefore, tested under three levels of inter-row spacing (i.e. 20, 25, and 30 cm) in randomized complete block design (RCBD) with three replications. The net plot sizes were varied according to the tested inter-row spacing i.e. 1.4 m  ×  1.6 m (2.24 m^2^), 1.3 m ×  1.6 m (2.08 m^2^), and 1.2 m ×  1.6 m (1.92 m^2^) for 0.2 m, 0.25 m, and 0.3 m row spacing, respectively. The central four rows were used for agronomic data collection and measurements. The distance between the experimental plots and blocks were kept at 0.5m and 1m apart, respectively. The central four rows were used for agronomic data measurements.

**Agronomic Management practices:** Prior to planting, the experimental sites have been plowing by oxen-driven, following the local tillage practice suitable for malt barley cultivation. In accordance with the specifications of the design, the field layout was prepared, and each treatment was assigned randomly to each experimental unit. Then the varieties were sown manually by drilling in rows at the recommended rate of 125 kg ha^−1^ on 18 June, and 26 July, 2020 at Kino and Miligebsa experimental sites, respectively. Nitrogen and phosphorus as Di-ammonium phosphate (DAP), and Urea (46-0-0), were uniformly supplied into all experimental plots at a rate of 46 kg ha^−1^ of nitrogen (N) and 20 kg ha^−1^ of phosphorus (P), under favourable weather to minimize nitrogen loss. Hence, due to its nature, nitrogen was split applied into two doses where the first half dose, together with all phosphorus, was applied during planting while the remaining half dose was applied 30 days after sowing (i.e. at the early tillering stage of malt barley) at both experimental sites. All plots at both sites were kept free of weeds, and the weeds were removed by hand weeding. Insecticide, herbicides, or fungicide was not applied, since there was no outbreak of insects or diseases during the entire cropping season. Harvesting was done manually using a hand sickle. Threshing was employed manually to separate the seeds.

### 2.1 Data collection and measurements

#### 2.1.1 Phenological and morphological traits

The data from the plant developmental stages, such as days to heading and physiological maturity were measured through visual observation. Phenological traits such as 50% days to heading, and 90% of days to physiological maturity were recorded from both experimental sites. These stages were determined using the Zadoks scale Zadoks et al. [1] when about 50% of the crop in the experimental plots achieved a particular stage. The 50% days to heading was recorded for about 50 percent of the particular experimental plot produces spikes above the sheath of the flag leaf Amare et al. [2]. Similarly, the data for 90% of physiological maturity was also recorded when the peduncles turned into golden yellow color Anteneh et al. [3].

#### 2.1.2 Agronomic traits

Quantitative agronomic traits were collected from both experimental sites. The traits viz., plant height (cm), number of seeds per spike, spike length (SL (cm)), and number of productive tillers, were measured from 10 randomly selected plants from the central four rows. Plant height was measured in each plot area from the base to the tip of the main stem during physiological maturity. Instruments such as a portable hanging weighing scale (Model: 23510S (SHFSB-0403)) was used to measure the aboveground biomass yield (t ha^−1^), and measuring tape were used to measure the spike length and plant stature. The mean data used in the analysis of agronomic traits was the average value of each malt barley variety from the two locations.

#### 2.1.3 Grain yield and 1000-seed weight

the grain yield of each plot was collected by considering the four central rows. The total seed yield was recorded by weighing the harvested seeds through a digital sensitive electronics balance, whereas the 1000-seed weight was obtained by counting, and weighing the harvested seeds by using the same tool. Both Grain yield and 1000-seed weight were adjusted to 12.5% grain moisture content as described by Badu-Apraku et al. [4].

$$Grain\;yield\;\left(kg\;ha^{-1}\right)=\ldots \times 100$$

**Where,**

**AMC** is the percent of actual grain moisture content (%) and **SMC** is the percent of standard grain moisture content

### 2.2 Statistical data analysis

The mean data of 10 randomly selected plants in each malt barley variety were used to determine a range of agronomic traits and the overall mean of each environment. All the collected agronomic and phenological data were subjected to statistical analysis using the general linear model (GLM) procedure of SAS 9.2 version [5]. To determine whether growing location-variety could be combined, we performed an ANOVA on the residuals of the combined analysis considering location, variety, and inter-row spacing, and their interactions, as a fixed factor. For the significant effects, means were separated using the least significance difference (LSD) at a 5% significance level. Duncan's Multiple Range Test (DMRT) was employed for comparison of the various interaction means presented in various graphs presented in this dataset article. The mean values were used to construct the graphs using excel graphing features.

## CRediT authorship contribution statement

**Amare Assefa Bogale:** Conceptualization, Methodology, Visualization, Data curation, Writing – original draft, Investigation, Formal analysis. **Anteneh Agezew Melash:** Writing – original draft, Validation, Formal analysis, Writing – review & editing. **Awoke Wasae Argaw:** Writing – original draft, Validation, Formal analysis, Writing – review & editing.

## Declaration of Competing Interest

The authors declare there is no known competing interest.
